# Glutathione Reductase Expression and Its Prognostic Significance in Colon Cancer

**DOI:** 10.3390/ijms25021097

**Published:** 2024-01-16

**Authors:** Marlena Brzozowa-Zasada, Adam Piecuch, Karolina Bajdak-Rusinek, Marek Michalski, Olesya Klymenko, Natalia Matysiak, Kamil Janelt, Zenon Czuba

**Affiliations:** 1 Department of Histology and Cell Pathology in Zabrze, Faculty of Medical Sciences in Zabrze, Medical University of Silesia in Katowice, 40-055 Katowice, Poland; 2 Department of Medical Genetics, Faculty of Medical Sciences in Katowice, Medical University of Silesia in Katowice, 40-055 Katowice, Poland; 3 Zabrze Silesian Nanomicroscopy Centre in Zabrze, Silesia LabMed—Research and Implementation Centre, Medical University of Silesia, 40-055 Katowice, Poland; 4 Department of Microbiology and Immunology, Faculty of Medical Sciences in Zabrze, Medical University of Silesia in Katowice, Jordana 19, 41-808 Zabrze, Poland

**Keywords:** colorectal cancer, glutathione system (GSH), glutaredoxin, chromosomal instability (CIN), ELISA, Western blot, prognostic factor, oxidative stress, redox homeostasis

## Abstract

Maintaining a balanced redox state within cells is crucial for the sustenance of life. The process involves continuous cytosolic disulfide reduction reactions to restore oxidized proteins to their reduced thiol forms. There are two main cellular antioxidant pathways—the thioredoxin (Trx) and glutathione (GSH)/glutaredoxin (Grx) systems. In the GSH/Grx system, glutathione reductase (GR; GSR) catalyses the reduction of GSH disulfide (GSSG) to its sulfhydryl form (GSH), which can then further reduce oxidized Grxs. GR is an essential enzyme that helps in maintaining the supply of reduced glutathione-GSH, which is a significant reducing thiol found in most cells and known for its antioxidant properties. Therefore, it can have a significant impact on cancer development. To investigate this further, we performed an immunohistochemical analysis of GR protein expression in colon adenocarcinoma samples collected from patients with primary colon adenocarcinoma (stage I and II) and patients with metastasis to regional lymph nodes (stage III). The results of our study revealed a significant relationship between the immunohistochemical expression of GR and tumour histological grade, depth of invasion, regional lymph node involvement, staging, and PCNA immunohistochemical expression. It was found that 95% of patients with stage I had low levels of GR expression, whereas 89% of patients with stage III had high levels of immunohistochemical expression. A high level of expression was also detected in the patients with stage II of the disease, where almost 63% were characterized by a high expression of GR. The Western blot method revealed that the highest level of expression was found in the LS 174T cell line, which corresponds to stage II. The results of our study indicate that the immunohistochemical expression of GR may act as an independent prognostic factor associated with colon adenocarcinoma patients’ prognosis.

## 1. Introduction

Colorectal cancer is a type of cancer that affects the colon as well as the rectum. It is the second most common cancer among women and the third most common among men. Colorectal cancer is also responsible for the fourth highest number of cancer-related deaths worldwide, accounting for 9.2% of all such deaths [1]. The survival rates for this cancer for five years and ten years are 65% and 58%, respectively. It is important to note that men are at a higher risk of developing this malignancy and have a 25% higher mortality rate than women [2]. Extensive screening for colorectal cancer has significantly reduced its incidence and mortality by detecting and removing precancerous adenomas at an early stage. However, the incidence of colorectal cancer is increasing in young adults due to many factors, such as a westernized diet, chronic stress, and lack of physical activity. This is concerning, as early detection and treatment are crucial in preventing the development and progression [3,4].

It should be pointed out that colorectal cancer results from multiple genetic events, mainly defects in chromosome segregation, telomere maintenance, and the DNA damage response, collectively known as chromosomal instability (CIN). This genetic instability is responsible for the majority of cases of colorectal cancer [5,6]. An excess of reactive oxygen species (ROS) and nitrogen species over antioxidant defences causes DNA damage through oxidative stress [7]. An increase in ROS level may lead to redox imbalance and cause tumour initiation and progression by activation of redox-responsive signalling cascades that promote cell growth [8]. Maintaining a balanced redox state within cells is crucial for the sustenance of life. This process involves continuous cytosolic disulfide reduction reactions to restore oxidized proteins to their reduced thiol forms. This is where the pentose phosphate pathway (PPP) comes into play, which oxidizes glucose to generate NADPH which acts as an electron donor for the two main cellular antioxidant pathways—the thioredoxin (Trx) and glutathione (GSH)/glutaredoxin (Grx) systems [9,10]. In the GSH/Grx system, glutathione reductase (GR; GSR) catalyses the reduction of GSH disulfide (GSSG) to its sulfhydryl form (GSH), which can then further reduce oxidized Grxs [11]. GSH can directly scavenge certain free radicals and ROS such as hydroxyl radical, lipid peroxyl radical, hypochlorous acid, peroxynitrite, and hydrogen peroxide [12,13,14]. Acevedo-Leon’s research has shown that there are significant differences in the levels of GSH and GSSG in patients with colorectal cancer when compared to a healthy control group. In CRC patients, GSH levels decreased by over 50%, while GSSG levels increased by over 140%, resulting in a significant elevation in the GSSG/GSH% serum ratio. This suggests a marked change in the redox state of the CRC patients, indicating a shift towards oxidation [15]. It is important to note that oxidative stress (OS) is not a unique mechanism in cancer progression in colorectal cancer, as other gastrointestinal (GI) diseases are also affected [16,17,18,19,20]. Ki et al. conducted a study that demonstrated that the mRNA and protein expression of GSH, the catalytic subunit of GCL (GCLC) and GSS were significantly increased in colon cancer cell lines including Caco 2, SNU 407, SNU 1033, HCT 116, and HT 29, compared to the normal colon cell line FHC. Additionally, GSH expression levels were found to be elevated in tumour tissue compared to adjacent normal tissue in 9 out of 15 patients with colorectal cancer. Immunohistochemical studies also showed that GCLC and GSS were higher in colorectal cancer tissue than in normal mucosa. Given that GSH and GSH-metabolising enzymes are found at increased levels in colon tumours, they could be clinically useful biomarkers for colon cancer and targets for anticancer therapy [21].

GR is an essential enzyme that helps in maintaining the supply of reduced glutathione-GSH, which is a significant reducing thiol found in most cells and known for its antioxidant properties. Some studies have attempted to define GR and other antioxidant enzymes as plasma or serum biomarkers that might predict the risk of colorectal cancer related to oxidative stress damage [22,23,24,25,26,27,28]. Moreover, Gaya-Bover et al. and Strzelczyk et al. analyse antioxidant protein levels including GR in both tumour and non-tumour adjacent tissue from colorectal cancer patients [29,30]. Unfortunately, the studies conducted so far have not been able to determine whether GR has any clinical or prognostic significance in colorectal cancer. Therefore, our study aims to determine the immunohistochemical expression of GR protein in samples of colon adenocarcinoma. We focused on patients with stage I, II, and III disease. It is important to note that while most patients with colorectal cancer are diagnosed at an advanced stage, there is a need to search for markers for early diagnosis and treatment of patients. Therefore, the choice of our study group seems justified. The results of the immunohistochemical expression of GR protein were correlated with the clinical data of the patients and the patient’s survival time. In addition, we wanted to verify the localization of the GR protein in tumour tissue, which probably could be the basis for future studies associated with the development of targeted cell therapy. Given that cancer tissue is a very heterogeneous environment with cancer cells but also with cells of cancer microenvironment, we also decided to carry out in vitro studies using cell lines to confirm the expression of GR protein strictly only in cancer cells without any exposition to signals from other parts of the tumour microenvironment as is the case for tissue in vivo. We utilized Western blot techniques for this purpose. The results of these studies will serve as a basis for further studies in which we intend to perform molecular in vitro studies related to *GR* gene expression. At this stage of our studies, we would like to verify whether the results obtained using immunohistochemical techniques in patients and samples of colon adenocarcinomas correlate with those obtained in vitro in the corresponding cell lines from stage I, II and III patients. Importantly, our study was complemented by an ELISA analysis of GR serum levels in patients. These data were also correlated with patient survival and clinical data.

## 2. Results

### 2.1. A Description of the Studied Group

Our study involved 143 patients, with 64 being men and 67 being women. The average age of the participants was 65 years, ranging from 55 to 77 years. Among all the cases, 61 (46.56%) had cancers on the right side of the colon, while 70 (53.44%) had cancers on the left side. The histological grades of differentiation were classified into three categories: G1 had 21 cases (16.03%), G2 had 69 cases (52.67%), and G3 had 41 cases (31.30%) (Table 1).

**Figure 1:**
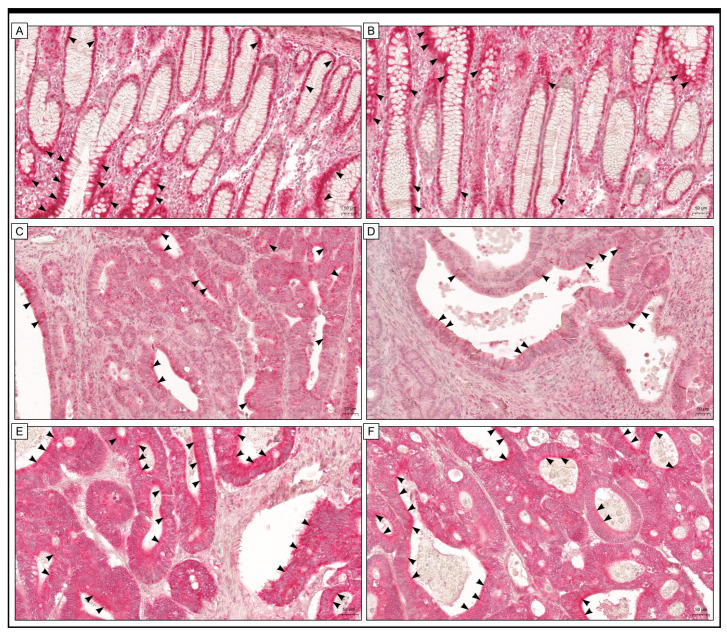
The immunohistochemical expression of GR was detected in adjacent non-cancerous tissue margins (**A**,**B**) and colon adenocarcinoma tissue (**C**–**F**). Black arrows show examples of cells in which immunohistochemical expression of the protein was detected, and a positive staining reaction (in red) indicates GR protein expression is visible. The scale bar is 50 µm for (**A**–**F**).

The immunohistochemical expression of GR protein was observed in both cancer cells and stromal cells in the colon adenocarcinoma samples. Moreover, the expression was also detected in cells of non-pathological colon mucosae. Out of the group being studied, 89 samples of colon adenocarcinoma (which is 67.94%) showed high levels of GR protein expression when examined immunohistochemically, while 42 samples (which is 32.06%) had low levels of immunoreactivity (Figure 1).

### 2.2. Clinically Relevant Parameters (as Independent Variables) Correlated with GR Immunohistochemical Expression

The next step was to compare the results of the immunohistochemical analysis to the clinicopathological features (as independent variables) of patients and their survival rates. It was found that GR expression had a significant correlation with the histological grade of the tumour (*p* = 0.007, Chi^2^ test). The high levels of GR protein expression were found in 9 samples (42.86%), 54 samples (78.26%), and 26 samples (63.41%) of the G1, G2, and G3 tumours, respectively. On the other hand, a low level of immunohistochemical expression of GR protein was revealed in 12 samples (57.14%), 15 samples (21.74%), and 15 samples (36.59%) of the G1, G2, and G3 tumours, respectively. Furthermore, GR protein expression was related to the depth of invasion (T) (*p* < 0.001, Chi^2^ test). Among T1/T2 patients, a high level of immunohistochemical reaction was observed in 11 patients (34.38%), while 21 patients (65.63%) had a low level of expression of this protein. For T3 patients, 63 (80.77%) had a strong immunohistochemical reaction for GR protein, while 15 (19.23%) had a low level of immunohistochemical expression. According to the study, the T4 group had 15 patients (71.43%) with high expression of GR, while the T4 group had 6 patients (28.57%) with low expression. It is worth noting that there was a significant association between GR expression and regional lymph node metastasis (*p* < 0.001, Chi^2^ test). In patients characterised as N0, 22 (40.74%) had high GR immunohistochemical expression, while 32 (59.26%) had low immunoreactivity. On the other hand, among patients with N1/N2 status, 67 (87.01%) had high GR expression, while 10 (12.99%) had low GR immunoreactivity (*p* < 0.001; Chi^2^ test). In patients with stage I of the disease, 1 subject (4.55%) showed high levels of GR expression, while 21 (95.45%) had low expression. For those with stage II of the disease, 22 patients (62.86%) had high GR expression, while 13 (37.14%) had low expression. In patients with stage III of the disease, the expression levels varied, with 66 individuals (89.19%) having high expression and 8 (10.81%) having low expression (*p* < 0.001, Chi^2^ test) (Table 2).

The study found a close correlation between GR expression and PCNA antigen expression (*p*-value < 0.001, Chi^2^ test). GR protein was significantly upregulated in 55.56% of samples with low PCNA immunoreactivity and 44.44% of samples with high PCNA immunoreactivity. When both PCNA and GR were treated as dependent variables, the analysis showed that 55.73% of the total group had high levels of PCNA and GR expression, and 15.27% had low levels of both. The number of patients with high levels of PCNA and low levels of GR expression (16.79% of the total) was similar to those with low levels of PCNA and high levels of GR expression (12.21% of the total) (Table 3).

### 2.3. Prognostic Role of GR Protein Expression in Colon Adenocarcinoma

**Figure 2:**
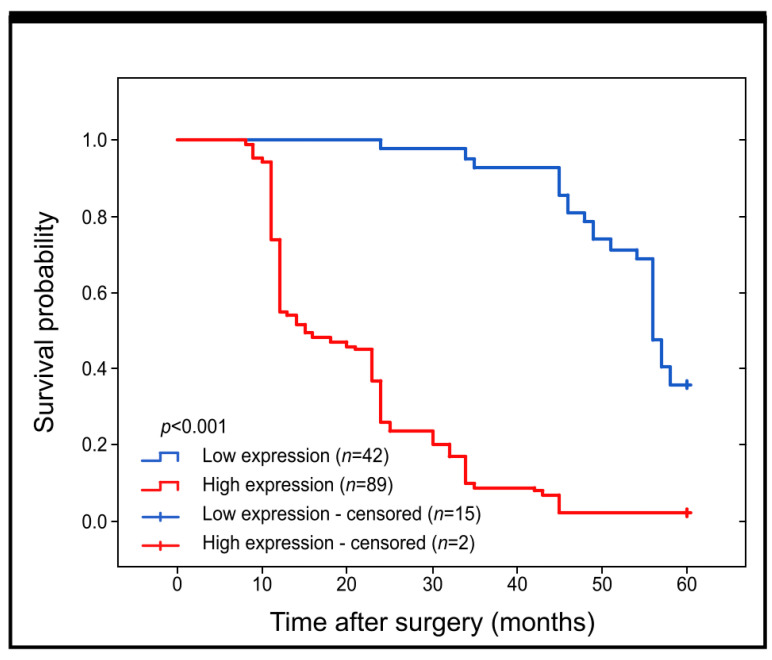
Kaplan–Meier curves for comparison of survival probability for patients with high and low levels of GR immunohistochemical expression in colon adenocarcinoma samples.

Our study aimed to determine whether the immunohistochemical expression of GR protein has any impact on the survival of patients with colorectal adenocarcinoma. We used Kaplan–Meier survival curves to analyse all samples. Our findings indicate that patients with low expression of GR had significantly better prognoses than those with high expression of this protein with Me Overall Survival = 56 and 15 for subjects with low and high expression of GR, respectively (log-rank, *p* < 0.001) (Figure 2).

**Figure 3:**
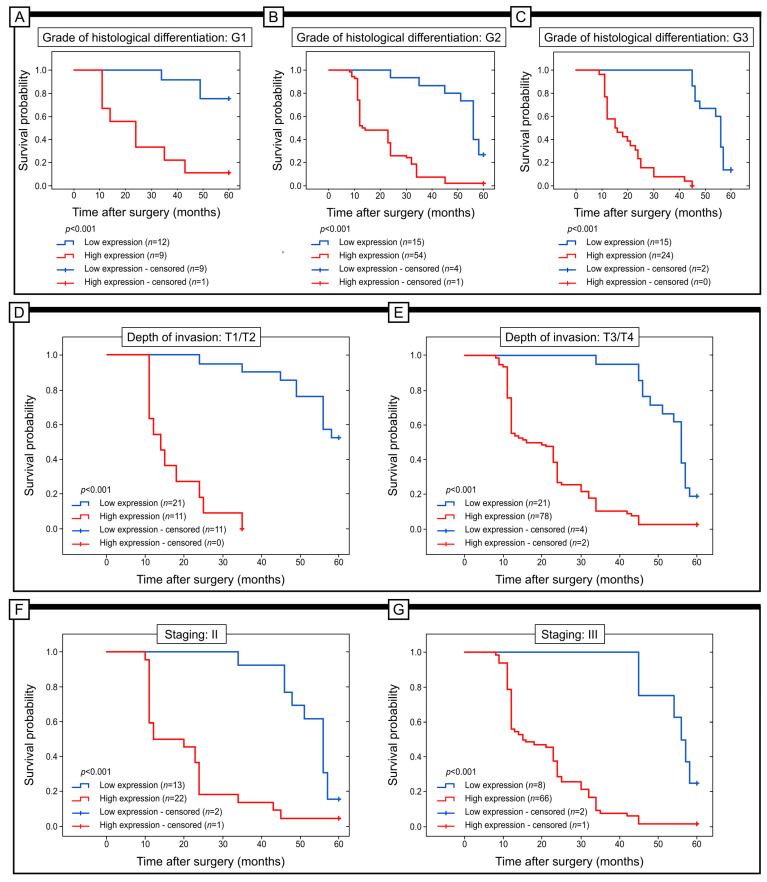

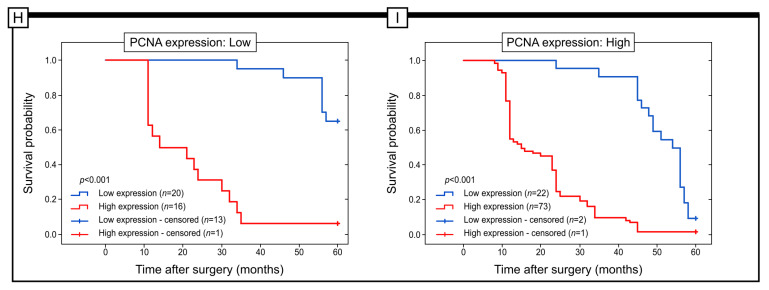
Kaplan–Meier curves were used to analyse the data on the immunohistochemical expression of GR protein in colon adenocarcinoma patients. The analysis was conducted through a log-rank test to compare patients with high versus low levels of GR expression. The results were presented for patients with different grades of differentiation (G1, G2 and G3) (**A**–**C**), depth of invasion (T1/T2 and T3/T4) (**D**,**E**), and different stages (II and III) (**F**,**G**) and immunohistochemical expression of PCNA (low and high expression) (**H**,**I**).

The study examined the relationship between the expression of GR and the survival of patients in different subgroups. Factors such as histological differentiation, depth of invasion, staging and PCNA immunohistochemical expression were taken into account. The results indicated that in patients categorized as G1, G2 and G2, lower levels of GR expression were associated with better prognosis with Me Overall Survival = 60, 56 and 56, respectively (log-rank test; *p* < 0.001). Comparable findings were observed in patients with T1/T2, T3 and T4 depth of invasion, as well as in those with I, II, and III stages of the disease with Me Overall Survival = 60, 56, 56, respectively, for all of these analysed factors. In these cases, the lower levels of GR expression were linked to better clinical outcomes (log-rank test; all *p* < 0.001) (Figure 3).

Our study examined various factors that may impact the survival of patients with colon adenocarcinoma. Through Cox regression analyses, we found that several factors including staging, histological differentiation grade, invasion depth, regional lymph node involvement, GR immunohistochemical expression, angioinvasion and PCNA expression were all significant prognostic factors. Specifically, we found that the grade of tumour differentiation and GR immunohistochemical expression were independent prognostic factors associated with the survival of patients with colon adenocarcinoma, as shown in Table 4 of our patient cohort data.

### 2.4. Immunofluorescence Staining

In our investigation, we wanted to examine the expression of GR protein in colon adenocarcinomas also by using the immunofluorescence technique as described in our previous studies [31,32] and a report published by Frithiof et al. [33]. Fifty tissue sections treated with anti-GR antibody and Dako Liquid Permanent Red chromogen (LPR) were chosen at random, including ten control samples, twenty-five samples of low expression as determined by immunohistochemistry, and twenty-five with high expression. This technique was used as an additional measure; however, the results were promising and suggested that the treatment of anti-GR antibody-stained tissue sections with LPR chromogen may be helpful in immunofluorescence analysis.

**Figure 4:**
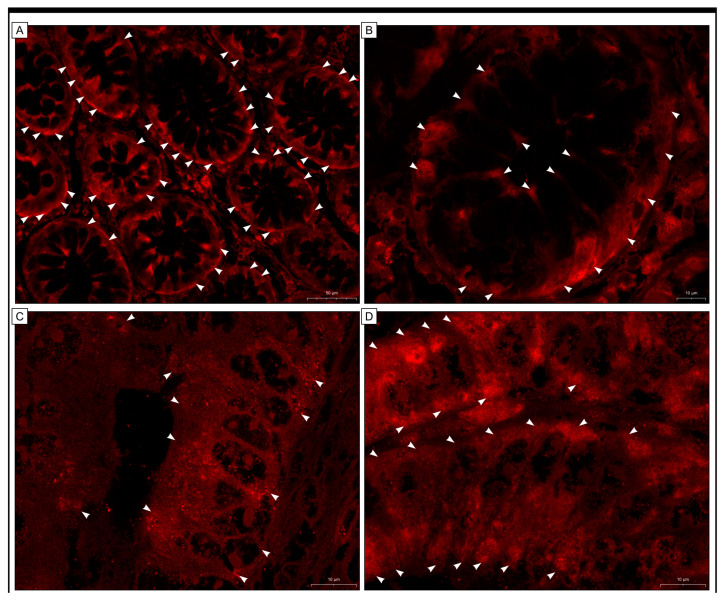

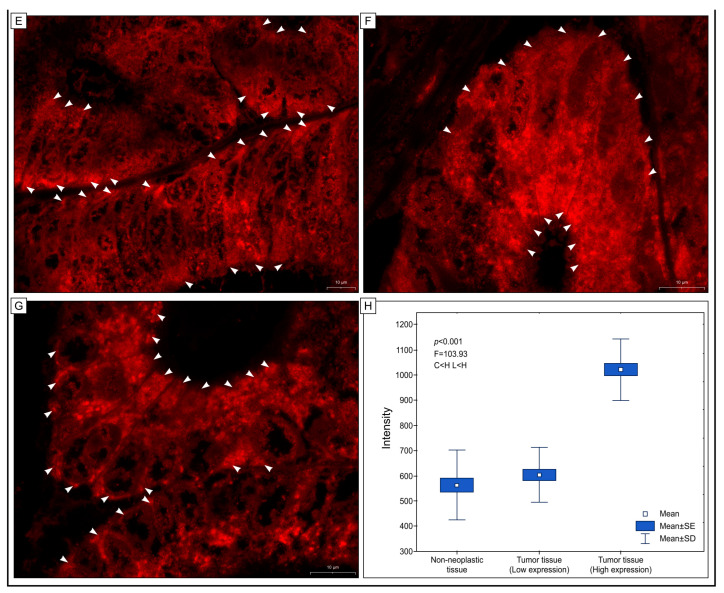
These figures illustrate the expression of GR protein in colon adenocarcinoma tissue (**C**–**G**) and non-carcinoma adjacent tissue (**A**,**B**) using immunofluorescence. A red fluorescent signal of fluctuating levels was seen in cells located within non-cancerous mucosa (**A**,**B**)—arrowheads show expression in the cytoplasm of colonocytes and cancer cells (**C**–**G**). In several cancer cells, the fluorescent signals showing the presence of GR were located in the cytoplasm of the apical parts of the cells, whereas in others, intense fluorescence was observed throughout the cytoplasm of the cells (arrowheads) or inside the nuclei (arrows). (**H**) ANOVA test results show differences in the intensity of the red signal representing the presence of GR between the groups tested: C < H, L < H—differences in intensity between the groups; C—colon tissue free of any pathological abnormalities, L—adenocarcinoma samples with low expression of GR, H—adenocarcinoma tissue exhibiting high expression of GR.

Zen 2 (blue edition) software was used to quantify GR expression levels in both normal and tumoral tissues. We observed the fluorescent staining of different levels of intensity in both cancerous and normal mucosal cells. Some cancer cells exhibited the fluorescent signal in the apical cytoplasmic regions, while other cells displayed strong fluorescence throughout the cytoplasm of the cells. In some cancer cells, the signal was also detected within the nucleus (Figure 4).

### 2.5. Intracellular Localization of GR Protein by the Method of Immunogold Labelling with the Use of Transmission Electron Microscopy (TEM)

**Figure 5:**
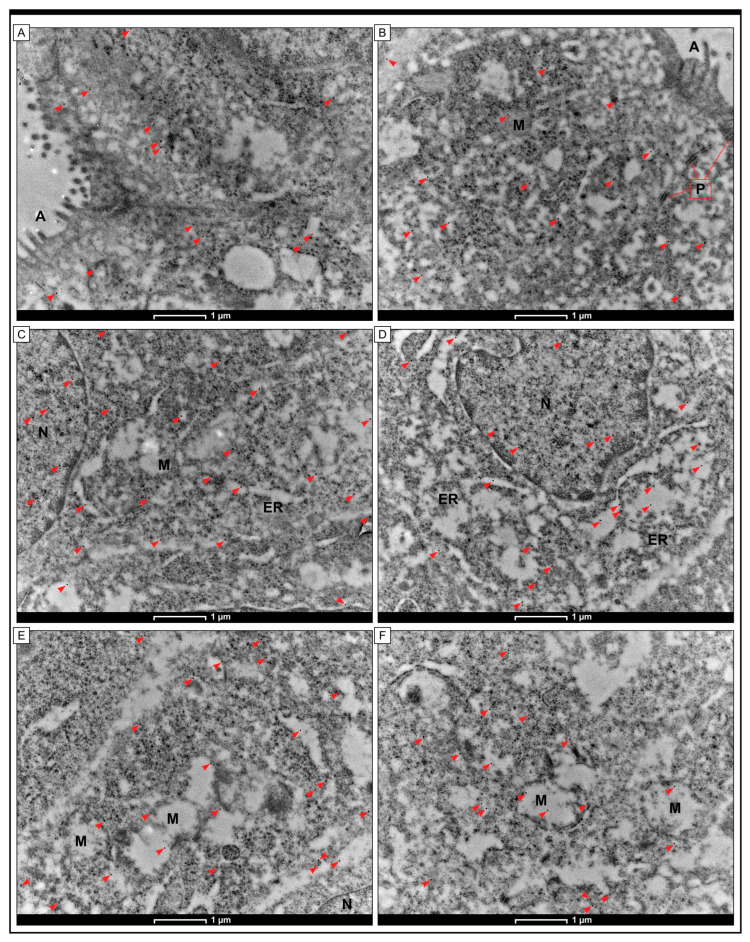
The presence of GR was confirmed in cells from healthy mucosa, where they were visible in the apical parts of the cells, mostly within the mitochondria (**A**,**B**)**.** In cancer cells, the localisation of the GR antigen was mainly associated with the cytoplasm and the nucleus (N). In the nucleus of neoplastic cells, granules indicating the presence of GR were found to be loosely distributed in the nuclear stroma and close to the nuclear membrane (**C**,**D**). In the cytoplasm, electron-dense black granules associated with the presence of GR were mainly localised in the mitochondria (M), where their presence was seen close to the mitochondrial membrane or in the matrix. Their presence was also detected in the cisternae of the endoplasmic reticulum (ER) (**E**,**F**). The scale bar is 1 µm for (**A**–**F**). Red arrowheads indicate the presence of electron-dense granules in cellular compartments, consistent with GR antigen.

The GR protein was identified in colon adenocarcinoma cells by immunogold labelling. At the level of TEM, it was found that black granules, consistent with the GR protein, were located in the cytoplasm and nucleus of cancer cells. In addition, electron-dense black aggregates were observed inside the mitochondria and cisterns of the rough endoplasmic reticulum. There were also scattered black granules observed in the cytoplasm of apical cells as well as in the plasma membrane of human colon cells obtained from non-cancerous specimens (Figure 5).

### 2.6. GR Protein Expression in Colorectal Cancer Cell Lines Detected by the WB Method

As cancer tissue is a complex environment consisting of cancer cells and cells in the cancer microenvironment, we decided to conduct in vitro studies using colorectal cancer cell lines. This approach enabled us to confirm the expression of GR protein solely in cancer cells without any exposure to signals from other parts of the tumour microenvironment, unlike in vivo tissue. To achieve this, we utilized the Western blot technique. The results of these studies will provide a foundation for further molecular in vitro studies concerning *GR* gene expression. Currently, we need to compare the findings obtained from immunohistochemical techniques used on patients and samples of colon adenocarcinomas with the ones obtained from in vitro studies on cell lines of stage I, II and III patients.

**Figure 6:**
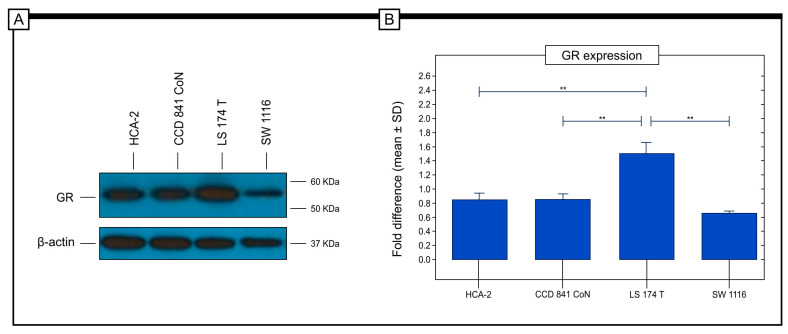
In vitro study of the expression of GR in colorectal cancer cell lines that represent a different type of colorectal cancer. The analysis revealed that the LS 174T cell line had the highest level of GR protein expression. A similar level of GR expression was observed in the SW1116 cell line, HCA-2 cell line and CCD 841 CoN (**A**). Significant differences in the levels of GR protein expression were observed between the HCA-2 cells and the LS 174T, between CCD 841 CoN and LS174T, and between SW 1116 and LS 174T. Statistical significance was determined using an independent-sample *t*-test. Data are shown as mean ± SD of values of three measurements in each group. In all figures, *p*-value of statistical significance is ** *p* < 0.01 (**B**).

The protein expression levels in cancer cell lines were assessed using the Western blot technique in vitro. The results showed that among the cancer cell lines, the LS 174 T (Stage II; Duke B) cell line had the highest level of GR protein expression. On the other hand, SW 1116 (stage I; Duke A), HCA-2 (stage I; Duke C) and CCD 841 CoN cells had similar levels of expression. Statistical analysis revealed significant differences in GR protein expression between the HCA-2 and LS 174T cell lines, between CCD 841 CoN and LS 174 T, and between LS 174T and SW 1116 cell lines (refer to Figure 6).

### 2.7. Serum Level of GR in Patients

**Figure 7:**
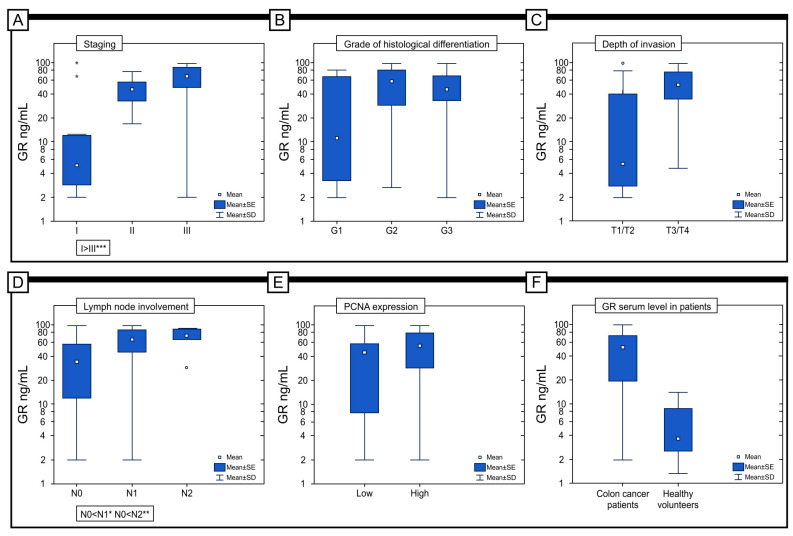
The serum GR levels in patients with colorectal adenocarcinoma were compared regarding staging (**A**), grade of histological differentiation (**B**), depth of invasion (**C**), lymph node involvement (**D**), and immunohistochemical expression of PCNA protein (**E**). (**F**) The concentration of the serum level in patients with colorectal adenocarcinoma and control subjects. * *p* < 0.05, ** *p* < 0.01, *** *p* < 0.001, °: statistical outliers.

The concentration of GR in the serum of patients with colon adenocarcinoma (M = 47.53 ng/mL; Me = 51.49 ng/mL) was significantly lower than in the serum of healthy volunteers (M = 5.46 ng/mL; Me = 3.65 ng/mL) (*p* < 0.001) (Figure 7F). In addition, there was a statistically significant difference in the level of GR in serum between patients with different stages of the disease, with the highest level of GR detected in patients with stage III disease (M = 63.33 ng/mL; Me = 67.48 ng/mL) and the lowest level detected in patients with stage I disease (M = 17.42 ng/mL; Me = 5.00 ng/mL) (*p* < 0.001). In the case of histological differentiation, the highest level was found in patients with G2 (M = 53.25 ng/mL; Me = 57.63 ng/mL) and the lowest level was found in the G1 subject (M = 28.72 ng/mL; Me = 11.05 ng/mL) (*p* = 0.067). Importantly, significantly higher levels were found in patients with T3/T4 (M = 55.37 versus M = 24.02 ng/mL) (*p* = 0.001) and N2 disease (M = 71.39 versus 58.01 versus 35.34 ng/mL) (*p* = 0.001), respectively, for the criteria of depth of invasion (T) and lymph node involvement (N) (Figure 7A-E). In our study, we also tried to answer the question of whether the expression of GR in the tumour tissue correlates with the GR content in the serum of patients The study showed that a higher serum level of GR characterizes patients with a high expression of GR in the colon adenocarcinoma samples (M = 54.26 versus M = 38.89 ng/mL) (*p* = 0.032). 

**Figure 8:**
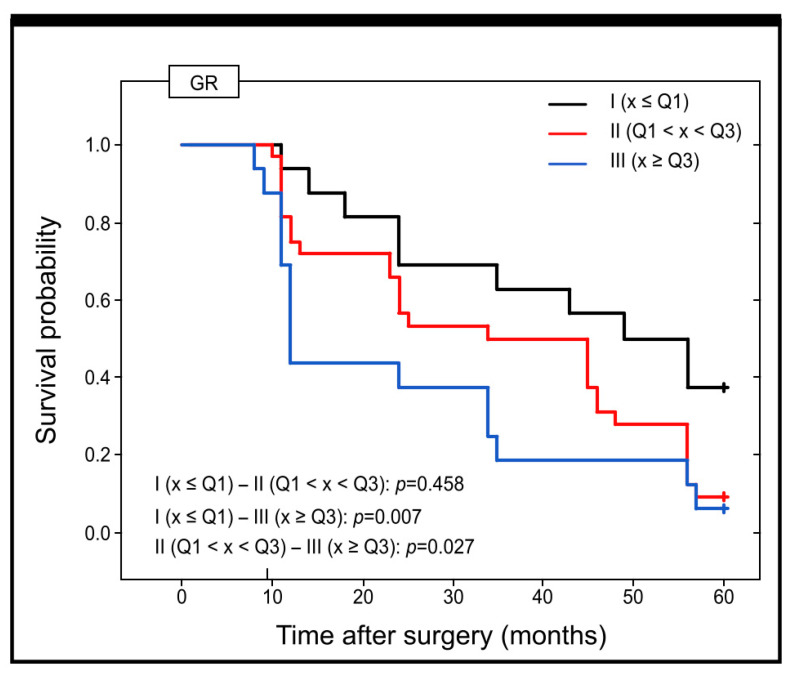
Survival probabilities and their transitions during the 60-month follow-up period for patients with different levels of serum GR in colorectal adenocarcinoma patients are shown in Kaplan–Meier curves.

With regard to the concentration of GR in the serum of the subjects and its prognostic importance, it is worth noting that there was a statistically significant difference in the predicted survival time between those patients with low levels of GR in the blood serum (group I) and with a medium concentration of GR in the serum (group II) (*p* = 0.048) and between patients with group I and those with a high concentration of GR in serum (III group) (*p* = 0.008) (Figure 8).

## 3. Discussion

ROS are produced as a result of the normal redox reactions that occur during cell metabolism. However, excessive amounts and inadequate elimination of ROS can lead to oxidative stress, which may cause severe metabolic dysfunctions and cell damage [34]. Elevated levels of ROS can react with biomolecules such as lipids, nucleic acids, and proteins, disrupting their normal function and contributing to chronic diseases, especially colorectal cancer [35,36,37]. Under normal physiological conditions, antioxidant systems and pro-oxidant systems are in a state of equilibrium. The protective antioxidant mechanisms include, e.g., the glutathione system. In cells, glutathione is mainly present in a reduced state (GSH) and, to a lesser extent, in an oxidized state (GSSG). This is due to the action of the glutathione reductase enzyme (GR), which reduces GSSG back to GSH [38,39]. Therefore, GR plays an important role in the regulation, modulation and maintenance of cellular redox homeostasis [40]. A lack of GR and GSH causes oxidative damage to the cell. Furthermore, many disorders are caused by GR and GSH deficiencies, including Alzheimer’s, Parkinson’s, liver and lung diseases, sickle cell anaemia, cancer and diabetes [41,42]. Several reports revealed that under in vitro conditions, inhibition of GR by 2-AAPA induced intracellular thiol oxidative stress, cell growth inhibition, cell cycle arrest and apoptosis in various cancer cells [43,44]. Li and colleagues conducted a study on murine melanoma cells and discovered that inhibiting GR using 2-AAPA resulted in thiol-induced oxidative stress. This was achieved by decreasing the GSH/GSSG ratio, which led to the inhibition of lung metastasis and subcutaneous growth of melanoma in vivo. In vitro experiments also showed that reducing GR activity had an inhibitory effect on various cellular processes of melanoma cells, including cell proliferation, colony formation, cell adhesion, migration, and invasion. Furthermore, the study found that inhibiting caused oxidative stress that blocked the epithelial-mesenchymal transition (EMT) by reducing the expression of vimentin, ERK1/2, and the transcription factor Snail. It also increased the expression of E-cadherin. The rearrangement of actin, which is a crucial element in cell motility, was also dependent on GR-induced oxidative stress, presumably through protein S-glutathionylation on actin [45]. Gopcevic et al. revealed that the activity of GR was found to be decreased in colorectal cancer patients compared to the control, and between TNM II and TNM III compared to the TNM IV stage of this malignancy. It is not clear whether the decrease in GR activity in stage III is a consequence of the reduction in glutathione supplies and/or NADPH availability or whether it is a result of inhibition on some other regulation level [25]. As mentioned earlier, the colon and the digestive tract are particularly exposed to ROS. Therefore, GR may play a special role in this type of cancer. Our study is the first to address the importance of the immunohistochemical expression of GR protein as a potential biomarker in the diagnosis of colorectal cancer, specifically adenocarcinoma. At this stage of our research, we have focused mainly on patients with stage I, II and III diseases, i.e., patients diagnosed with a primary tumour in the colon (I, II) and those with cancer metastases in the regional lymph nodes (III). Stage IV patients are a very complex and heterogeneous group that requires special and separate analysis, and we deliberately excluded them from our group at this stage of our research, but we wanted to examine the expression of GR protein also in this group of patients in the future. The results of our study seem very interesting. We revealed that the immunohistochemical expression of GR protein was detected not only in samples of colon adenocarcinoma but also in samples of non-pathological colon mucosa. These were supported by the immunofluorescence study with the use of a confocal microscope. Our research findings indicate a significant correlation between GR immunohistochemical expression and various factors related to tumour progression, such as tumour histological grade (G), depth of invasion (T), staging, lymph node involvement (N) and PCNA immunohistochemical expression. High immunohistochemical expression of GR protein was detected mainly in patients with a G2 grade of differentiation. In comparison to the G1 and G3 patients, high expression levels are the predominant feature in G2, as 78% of patients with this differentiation stage have high expression levels. Notably, 43% of G1 patients have high expression, while 63% of G3 patients have low expression. The results are also interesting when the depth of invasion is taken into account. Significantly higher levels of expression are seen in T3 and T4 patients, as well as in N2 patients. In the former, only 19% of patients have a low level of GR expression, while in the latter, as many as 71% have a high level of GR expression. If we look at stage N2, only 13% of patients have a low level of GR expression, while the rest have a high level of GR expression in samples of colon adenocarcinoma.

The modification in antioxidant enzyme activities observed in colorectal cancer patients could represent an index of oxidative stress and potential biomarkers associated with the patient’s prognosis [46,47,48,49]. For example, an increase in SOD activity, that transforms superoxide (O_2_^−^) to H_2_O_2_, and the decrease in CAT and GR, which cannot counteract the overproduction of H_2_O_2_, leads to the accumulation of the deleterious H_2_O_2_, thus shifting the redox balance. It has been reported that human cancer produces a large amount of H_2_O_2_ [50]. Some studies demonstrated that in colorectal cancer samples, the glutathione consumption and recycling system, Gpxs and GR, was downregulated in tumour tissue compared to non-tumour adjacent tissue. A downregulation of glutathione consumption and recycling system results in an accumulation of hydrogen peroxide. Thus, oxidative stress may increase, which could enhance the initiation and progression of cancer [28,29].

The results of our study showed a significant alteration in GR between stage I and stages II and III. With the progression from stage I to stage II, the size of the tumour increases. As the tumour expands, its centre becomes hypoxic [51]. In this case, high levels of expression predominate in stage III patients—as many as 89% of individuals have high levels of immunohistochemical expression, while in stage I patients, only about 5% have high levels of expression in the tumour tissue. Moreover, the high expression was also detected in stage II patients’ tissues. However, there was no statistical significance between the immunohistochemical expression of GR in patients with stage II and III. Several studies indicate that hypoxia can lead to a surge in anion superoxide production, even in cases where mitochondrial functionality is optimal. During stage II or III, where hypoxia may be more prevalent, an increase in GR expression was observed. As revealed by the studies, in stages II and III, higher levels of MnSOD/CAT and MnSOD/GPx ratio were also noted, which resulted in elevated hydrogen peroxide production and oxidative stress [29]. Significantly, the studies analysing GR levels in patients’ blood confirmed these results. In colon cancer patients, the highest serum levels were found in stage II and III patients, which was reflected in the survival curves. A higher level of GR in the serum has been associated with poor outcomes. It has been suggested that the increase in GR in the serum of patients with stage II and III colon adenocarcinoma may be a compensatory mechanism in response to high levels of oxidative stress [20]. In a separate study by Jelic and colleagues, an increase in the activity of antioxidant enzymes, including GR, was found to be correlated with a greater extent of oxidative damage and gene expression in locally advanced cervical carcinoma patients [52]. However, despite the high level of GR expression, this mechanism does not seem to be sufficient, as these patients have poor survival rates. This suggests that GR protein may be involved in the progression of colon cancer and its high level of expression could potentially be used as a biomarker to identify patients with a more aggressive form of the tumour.

Cell line studies showed that the LS 174T cell line exhibited the highest level of expression, whereas in the HCA-2 cell line, the expression of GR was similar to SW 1116 and CCD841 CoN cells. However, it should be noted that these results may be different from those obtained from the study with the use of tumour tissue, which is a complex structure. It should be mentioned that cancer tissue is composed of cancer cells and cells from the tumour microenvironment. In such a complex structure, a complex cellular network exists [53,54].

In the context of planned future molecular research, it seems to be important to know the intracellular localization of the GR protein. The results of our study showed that GR was present in the cytoplasm of cancerous cells. The gold granules that indicated the presence of GR protein were associated with the cell membrane, endoplasmic reticulum membranes, and mitochondria. In some cells, GR was also found in nuclei. As in yeast, human cells exhibit GR protein in mitochondria and cytosol, indistinguishable by Western blot [55]. Nuclear GR, Gpx, and GST probably contribute to antioxidant defence mechanisms, but the functions served by the localization of these antioxidants in nucleoli are less evident [56].

The reduced form of glutathione serves as a protective factor for cells, not only in terms of redox homeostasis but also in the face of the cytotoxic effects of chemical substances, including anticancer drugs. It has been suggested that glutathione and other antioxidant enzymes may be stimulated to counteract the increase in oxidative stress during chemotherapy treatment for colorectal cancer. One way that chemotherapy works is by increasing oxidative stress in cancer cells through the promotion or induction of reactive oxygen species, which can lead to apoptosis [57,58,59]. To make matters worse, the body’s antioxidant capacity is overstimulated in such a situation and cancer cells may develop chemoresistance, making chemotherapy less effective [60]. According to multiple reports, having high levels of GSH is linked to being more resistant to chemotherapy and radiation treatments. Conversely, reducing GSH levels could make cancer cells more susceptible to various types of programmed cell death and improve their sensitivity to chemotherapy [61]. In an in vitro study, intracellular GSH depletion made cancer cells more sensitive to oxidative stress, overcoming drug resistance and further improving the outcome of cancer therapy [62]. According to Hiang et al., patients who underwent chemotherapy had significantly higher levels of plasma GSH and GSSG after tumour resection compared to before the operation. These levels then decreased to levels similar to before the operation in the post-chemotherapy period. On the other hand, patients who did not receive chemotherapy had an unchanged plasma GSH level throughout the three different time points, but they gradually showed an increased plasma GSSG level from before the operation, after the operation, and through the post-chemotherapy period. In both pre- and post-resection periods, patients in the chemotherapy and non-chemotherapy groups had similar levels of oxidative stress indicators and GSH-related antioxidant capacities. However, after chemotherapy, patients in the chemotherapy group had significantly lower levels of plasma GSH and GSSG but had significantly higher plasma GPx and GR levels than patients in the non-chemotherapy group [63].

Lifestyle and dietary habits, such as stress, smoking, alcohol consumption, and high-fat or high-carbohydrate diets, can lead to the overproduction of ROS and cause oxidative stress if the antioxidant barrier is ineffective. In such cases, the metabolism of GSH plays a crucial role in antioxidative defence mechanisms. It protects cells from oxidative stress by reducing the disulfide bonds of cytoplasmic proteins to cysteines. GSH is then oxidized to glutathione disulfide (GSSG) [15]. Recent studies have shown that the use of antioxidants in patients with colorectal cancer can have both inhibitory and intensifying effects on tumour initiation and progression. Interestingly, increasing the amount of antioxidants in the diet does not generally improve the prognosis of cancer patients, as shown in epidemiological studies. Moreover, it has been found that oxidative stress increases in metastasizing cells and reduces distant metastasis, which offers new treatment possibilities for CRC patients. However, further research is needed in both in vitro models and clinical trials to investigate the potential benefits of antioxidants in CRC patients [64,65,66]. 

What about the challenges in translating our findings regarding GR expression into clinical practice? A major focus of translational research is the identification of biochemical molecules that can serve as clinical markers for colon adenocarcinoma patients. This is particularly important for tumours in the gastrointestinal tract, where markers must be reproducible, sensitive, and specific to meet the expectations of the clinical community. In order for a biomarker to be useful, it must first be validated as a predictor of disease, with considerations for specificity, sensitivity, and potential confounding factors. The sampling and analytical procedures used for validation must also take into account the constraints and non-invasiveness of sampling, the stability of potential biomarkers, and the simplicity and speed of the analytical method. While features of the cancer itself, such as nodal status (pN) and depth of primary tumour infiltration (pT), are important prognostic factors for cancer patients, the only reliable way to assess cancer stage is through a histopathological analysis of the removed tumour and surrounding tissue. However, new serum biomarkers, particularly those associated with oxidative stress, may be helpful in preoperative diagnosis, allowing for a non-invasive assessment of cancer progression. This could facilitate the selection of appropriate treatment and improve the survival rate of colorectal cancer patients. Our study is a first step towards further clinical trials focused on evaluating the diagnostic utility of redox biomarkers in a larger population of colon adenocarcinoma patients. The assessment of tumour progression may be aided by the determination of redox parameters. Assessing the relationship between oxidative stress intensity and colon adenocarcinoma survival seems appropriate. As we only analysed one enzyme associated with redox parameters, we cannot fully characterise the oxido-redox balance in our cohort of patients. Evaluating the relationship between the severity of oxidative stress and the clinical outcome of patients would be interesting and advisable in the future. 

Although our study presents some promising findings, it is important to acknowledge its limitations. The size of the cohort studied was limited, which may introduce selection bias and limit the generalizability of our results. To increase the sample size and gain a better understanding of the mechanism of GR activity, we recommend conducting future studies that employ in vitro molecular experiments. It would also be valuable to assess the redox status in the serum of patients and the examined cells. Conducting such studies would allow linking observed changes in the GSH system to disruptions in the redox homeostasis in patients with colorectal cancer. However, our work has some advantages. We carefully selected a group of patients without any accompanying diseases, which strengthens the validity of our findings. Furthermore, our study serves as a starting point for further clinical trials that assess the diagnostic utility of redox biomarkers in a larger population of colorectal cancer patients. In addition, we suggest conducting functional analyses in colorectal cancer cells, particularly those obtained from patients, to investigate how the manipulation of GR expression levels affects cellular behaviour.

## 4. Conclusions

We investigated the expression of the GR protein in colorectal adenocarcinoma tissue from patients with stage I, II and III colon adenocarcinomas of European origin (Poland). We used immunohistochemical and immunofluorescence techniques to perform this. Out of the group being studied, 89 samples of colon adenocarcinoma (67.94%) showed high levels of GR protein expression when examined immunohistochemically, while 42 samples (32.06%) had low levels of immunoreactivity. Our findings indicate that patients with a low expression of GR had significantly better prognoses than those with a high expression of this protein. Specifically, by the use of multivariate analysis, we found that the grade of tumour differentiation and GR immunohistochemical expression were independent prognostic factors associated with survival in our cohort of patients. The GR protein was identified in colon adenocarcinoma cells by immunogold labelling. At the level of TEM, it was found that black granules, consistent with the GR protein, were located in the cytoplasm and nucleus of cancer cells. In addition, electron-dense black aggregates were observed inside the mitochondria and cisterns of the rough endoplasmic reticulum. In colon cancer patients, the highest serum levels were found in stage II and III patients, which was reflected in the survival curves. A higher level of GR in the serum has been associated with poor outcomes. The in vitro tests showed that the LS 174T cell line exhibited the highest level of expression, whereas in the HCA-2 cell line, the expression of GR was similar to SW 1116 and CCD841 CoN cells.

## 5. Materials and Methods

### 5.1. Samples from Tumours and Patients

Colon tissue samples were collected from individuals with colon adenocarcinoma confirmed by histopathological examination during colon resection at Jaworzno Municipal Hospital and in Specialist Hospital in Zabrze from January 2014 to December 2017. Excluded from the CRC patient and control groups were individuals with systemic or autoimmune diseases such as insulin resistance, hypertension, coronary artery disease, thyroid, lung, kidney, liver, gastrointestinal, infectious diseases (HCV and HIV infection), and immune disorders. The study and control groups excluded smokers and patients who had taken antibiotics, glucocorticosteroids, non-steroidal anti-inflammatory drugs, vitamins, and nutritional supplements in the last 3 months. Additionally, only individuals who followed a standard diet were eligible for the study. The study excluded patients who had undergone preoperative radiotherapy or chemotherapy, had severe medical conditions or distant metastases, had inflammatory bowel disease, had tumour recurrence, or had a histopathological subtype other than adenocarcinoma. We followed a standardized protocol and obtained histopathological sections from each surgical specimen, including tumour fragments and adjacent tissue without tumour abnormalities. The specimens were preserved in formalin and then embedded in paraffin blocks. Later, the blocks were sectioned and stained with H&E to diagnose histopathology. We also examined the marginal tissue sections, and if any cancer cells were detected, we excluded the material from the study. The patients were followed up for 5 years to assess the prognostic significance of the GR protein.

### 5.2. Immunohistochemical and Immunofluorescence Staining

The specimens of colon adenocarcinoma and resected margins were fixed in formalin and embedded in paraffin blocks. Then, 4 µm thick sections were cut from these blocks and placed on Polysine slides. The sections were deparaffinized in xylene and rehydrated using a graded series of alcohol. In order to retrieve the antigenicity, the tissue sections were treated with microwaves in a 10 mM citrate buffer (pH 6.0) for 8 min each. The sections were treated with Glutathione reductase and PCNA antibodies GeneTex, GTX114199; 1:1000 and GTX100539, respectively; GeneTex. Irvine, CA, USA) at final dilutions of 1:700 and 1:600, respectively. To visualize protein expression, BrightVision (Cat. No. DPVB55HRP WellMed BV, ’t Holland 31, 6921 GX Duiven, The Netherlands) and Permanent AP Red Chromogen (Dako LPR from Agilent Technologies Code K0640; Santa Clara, CA 95051; USA) were used. Mayer’s haematoxylin was used to counterstain the nuclei. To analyse the results of immune histochemical staining, we followed the immunoreactive score used in previous publications [37,38]. The score was based on both the intensity and the number of cells with positive reactions. The intensity was graded as follows: 0 for no signal, 1 for weak, 2 for moderate, and 3 for strong staining. We assessed the frequency of positive cells semiquantitatively by evaluating the entire section, and each sample was scored on a scale of 0 to 4: 0 for negative, 1 for positive staining in 10–25% of cells, 2 for 26–50% of cells, 3 for 51–75% of cells, and 4 for 76–100% of cells. We then calculated a total score of 0–12 and graded it as follows: I for scores 0–1, II for scores 2–4, III for scores 5–8, and IV for scores 9–12. We considered grade I as negative and grades II, III, and IV as positive. Grades I and II represented low expression (no or weak staining), and grades III and IV represented high expression (strong staining). The evaluation was carried out by two independent pathologists, and any differences in opinion were resolved by consensus.

Sections were treated with anti-GR antibody and Dako Liquid Permanent Red (LPR) for immunofluorescence. Fluorescence of GR protein was observed using a confocal fluorescence microscope ((Zeiss LSM 980 with Airscan 2; Zeiss; Oberkochen, Germany) with excitation at 592 nm and emission between 574 and 735 nm using TexRed filter kits. Zeiss Zen 3.4 (blue edition) version 3.4.91.00000 (Zeiss; Germany) was used to measure the intensity of GR expression in both non-neoplastic and tumor tissue.

### 5.3. Immunogold Electron Microscopy

In this study, the samples were fixed by immersing them in a solution of 4% paraformaldehyde in 0.1 M phosphate-buffered saline (PBS) at room temperature for two hours. After that, they were rinsed several times in PBS. Next, the specimens were dehydrated in a series of graded ethanol and infiltrated for 30 min on ice in a mixture of 2 parts ethanol to 1 part LR White and a mixture of 1 part ethanol to 2 parts LR White. Following the infiltration with pure LR White, ultrathin sections (70 nm) were cut using an RMC Boeck-eler Power Tomo PC ultramicrotome equipped with a 45° diamond blade (Diatom AG, Biel, Switzerland). The ultrasections were then immunolabeled and placed onto nickel grids coated with Formvar. Beforehand, the sections on the grids were pre-incubated for 30 min by floating on drops of 50 mM NH4 Cl in PBS, followed by 30 min of blocking on drops of 1% BSA in PBS. The grids were treated overnight (16–18 h) at a temperature of 4 °C with a 1:20 dilution of primary anti-Glutathione reductase antibody (GeneTex, GTX114199) in BSA. The bound antibodies were localized by incubating the sections with immunogold-conjugated goat anti-mouse IgG 15 nm (BBInternational BBI Solutions, Sittingbourne, UK) diluted 1:100 for 1 h. Afterwards, the grids were rinsed with PBS drops (five changes, 5 min each) and water (three changes, 3 min each) before staining with 0.5% aqueous uranyl acetate. The main antibody was not included in the control group. After preparation, the grids were air-dried and examined under a TECNAI 12 G2 Spirit Bio Twin FEI Company transmission electron microscope at 120 kV. The images were captured using a Morada CCD camera (Gatan RIO 9, Pleasanton, CA, USA).

### 5.4. Colorectal Cancer Cell Lines

For the experiments, three different colorectal cancer cell lines were used—HCA-2 (Duke C), LS 174T (Duke B), and SW 1116 (Duke A), along with a normal epithelial cell line known as CCD 841 CoN. SW 1116, LS 174T and HCA-2 cell lines were provided by ATCC (American Type Culture Collection ATCC^®^, Old Town Manassas, VA, USA). The HCA-2 line is a donation from Prof Magdalena Skonieczna of the Silesian University of Technology. The following cell lines were chosen to correspond as closely as possible to the different stages of cancer and to reflect the cancer cells at each stage of the disease, i.e., the SW 1116 line corresponds to Duke A or stage I, the Lg line corresponds to Duke B or stage II and the HCA-2 line corresponds to Duke C or stage III of the disease. Line SW 1116 (ref CCL233) was isolated from a Caucasian man aged 73. Line LS 174T (ref CL 188) is a cell with epithelial morphology that was isolated from the colon of a 58-year-old Caucasian woman with adenocarcinoma of the colon. This cell line was deposited by Northwestern University and can be used for cancer research. The line CCD 841 CoN the cells that have been isolated from normal human colon tissue. The cells resemble epithelial cells but do not contain keratin, and there is no clear evidence of their epithelial origin.

To promote optimal cell growth, we utilized specific culture media for each cell line. For CCD 841CoN and LS 174T cell lines, we used Eagle’s minimum essential medium (EMEM) (ATCC 30-2003), while Dulbecco’s modified Eagle’s medium/Nutrient Mixture F-12 Ham (DMEM) from Sigma-Aldrich D8437(Saint Louis, MO, USA) was used for the SW 1116 and HCA-2 cell lines. Additionally, both media were supplemented with 10% foetal bovine serum ((FBS), ATCC 30-2020) and 1% penicillin–streptomycin–neomycin stabilized solution (Sigma-Aldrich P4083) for optimal growth conditions.

### 5.5. GR Expression in Colorectal Cancer Cell Lines

An amount of 8 µg of protein was separated by 15% sodium dodecyl sulfate-polyacrylamide gel electrophoresis (SDS-PAGE) and transferred to nitrocellulose membranes for 2 h using the Tetra Cell-Blot (Biorad, Müchen, Germany) with 1× blotting buffer (20 mM Tris, 150 mM glycine, 20% methanol, pH 8.3). Western blot protein detection was performed with rabbit anti-Glutathione Reductase (GeneTex, GTX114199; 1:1000), mouse anti-β-Actin (R&D SYSTEM, MAB8929; 1:5000; Minneapolis, Minnesota, USA) antibodies in 5% dry milk/TBS/Tween, followed by species-specific secondary HRP-coupled antibody incubation (Invitrogen, Karlsruhe, Germany, 1:25,000). Protein bands were visualized using SuperSignal© Western Blot Enhancer (Thermo Scientific, Karlsruhe, Germany, 46640), SuperSignal© West Femto Maximum Sensitivity Substrate (Thermo Scientific, Karlsruhe, Germany, 34095) and AmershamHyperfilm ECL (GE Healthcare, Freiburg, Germany, 28906839) films.

### 5.6. Serum Level of GR in Colon Adenocarcinoma Patients

The study group consisted of 73 colon adenocarcinoma patients (40 males and 33 females, aged 36–89 years) and 20 healthy volunteers as the control group. Excluded from the CRC patient and control groups were individuals with systemic or autoimmune diseases such as insulin resistance, hypertension, coronary artery disease, thyroid, lung, kidney, liver, gastrointestinal, infectious diseases (HCV and HIV infection), and immune disorders. The study and control groups excluded smokers and patients who had taken antibiotics, glucocorticosteroids, non-steroidal anti-inflammatory drugs, vitamins, and nutritional supplements in the last 3 months. Additionally, only individuals who followed a standard diet were eligible for the study. Blood samples were taken from colon adenocarcinoma patients before the treatment and frozen at −80 °C. The serum level of GR was assessed with the enzyme-linked immunosorbent assay (ELISA) according to the manufacturer’s instructions (SEB314Hu96 Tests Enzyme-linked Immunosorbent Assay Kit for Glutathione Reductase (GR); Cloud-Clone Corp., Wuhan, China). For statistical analysis, all subjects were divided into three groups according to the quartile range of GR serum levels in the total group (Table 5).

### 5.7. Statistical Analysis

In this study, we analysed the relationship between GR immunohistochemical ex-pression and relevant clinical parameters using Statistica 9.1 software developed by StatSoft in Krakow, Poland. To evaluate all numerical variables, we used the statistical measures of median and range. We assessed the relative characteristics of the groups studied using both the chi-squared test (Ch^2^ test) and Yates’s chi-squared test (Chi^2^_Yatesa_ test). In the case of the correlation between GR and PCNA treated as dependent variables, we used the McNemara test. The study assessed the relationship between Grx1 expression and patient survival using Kaplan–Meier analysis and the log-rank test. Statistical significance was set at *p* < 0.05. A similar test was used to assess the probability of colon adenocarcinoma patients in relation to the GR serum level. For the analysis of the relationship between clinical parameters and the GR serum level, the Wilcoxon paired *t*-test was used.

## Figures and Tables

**Table 1:** Characteristics of patients included in the study (n = 131).

		N (Number of Cases)	%
Gender	Females	67	51.15
	Males	64	48.85
Age (years)	≤60 years	51	38.93
	61–75 years	42	32.06
	>75 years	38	29.01
	M ± SD	64.56 ± 13.27	
	Me [Q1–Q3]	65 [age range: 55–77]	
	Min–Max	33–89	
Grade of histological differentiation	G1	21	16.03
	G2	69	52.67
	G3	41	31.30
Depth of invasion	T1	13	9.92
	T2	19	14.51
	T3	78	59.54
	T4	21	16.03
Regional lymph node involvement	N0	54	41.22
	N1	45	34.35
	N2	32	24.43
Location of tumour	Proximal	70	53.44
	Distal	61	46.56
Angioinvasion	No	27	20.61
	Yes	104	79.39
PCNA immunohistochemical expression	Low	36	27.48
	High	95	72.52
Staging	I	22	16.79
	II	35	26.72
	III	74	56.49

**Table 2:** Correlations between the immunohistochemical expression of GR protein and clinicopathological characteristics in colon adenocarcinoma patients.

		The Immunoexpression Level of GR				*p*-Value
		Low		High		
Age (years)	≤60 years	14	(27.45%)	37	(72.55%)	*p* = 0.548
	61–75 years	16	(38.1%)	26	(61.9%)	
	>75 years	12	(31.58%)	26	(68.42%)	
Gender	Females	20	(29.85%)	47	(70.15%)	*p* = 0.579
	Males	22	(34.38%)	42	(65.63%)	
Grade of histological differentiation	G1	12	(57.14%)	9	(42.86%)	*p* = 0.007
	G2	15	(21.74%)	54	(78.26%)	
	G3	15	(36.59%)	26	(63.41%)	
Depth of invasion	T1/T2	21	(65.63%)	11	(34.38%)	*p* < 0.001
	T3	15	(19.23%)	63	(80.77%)	
	T4	6	(28.57%)	15	(71.43%)	
Regional lymph node involvement	N0	32	(59.26%)	22	(40.74%)	*p* < 0.001
	N1/N2	10	(12.99%)	67	(87.01%)	
Location of tumour	Proximal	23	(32.86%)	47	(67.14%)	*p* = 0.834
	Distal	19	(31.15%)	42	(68.85%)	
Angioinvasion	No	12	(44.44%)	15	(55.56%)	*p* = 0.188
	Yes	30	(28.85%)	74	(71.15%)	
PCNA immunohistochemical expression	Low	20	(55.56%)	16	(44.44%)	*p* < 0.001
	High	22	(23.16%)	73	(76.84%)	
Staging	I	21	(95.45%)	1	(4.55%)	*p* < 0.001
	II	13	(37.14%)	22	(62.86%)	
	III	8	(10.81%)	66	(89.19%)	

**Table 3:** Correlations between the expression of GR protein and PCNA protein.

		The Immunoexpression Level of GR				*p*-Value
		Low		High		
PCNA Immunoexpression	Low	20	(15.27%)	16	(12.21%)	*p* = 0.860
	High	22	(16.79%)	73	(55.73%)	*p* < 0.001

**Table 4:** Cox regression analyses.

Prognostic Parameter	Univariate Analysis			Multivariate Analysis		
	HR	95% CI	*p*-Value	HR	95% CI	*p*-Value
Gender	1.033	0.715–1.491	0.864	–	–	–
Age	0.984	0.782–1.237	0.885	–	–	–
Staging (S)	2.093	1.598–2.741	<0.001	1.217	0.705–2.100	0.480
Grade of histological differentiation (G)	1.392	1.074–1.805	0.012	1.584	1.068–2.350	0.022
Depth of invasion (T)	1.397	1.122–1.739	0.003	0.752	0.553–1.023	0.070
Nodal status (N)	1.633	1.289–2.069	<0.001	0.799	0.545–1.173	0.252
Localisation	0.984	0.681–1.423	0.931	–	–	–
GR expression	8.061	4.890–13.290	<0.001	9.704	4.911–19.173	<0.001
Angioinvasion	2.076	1.235–3.491	0.006	1.223	0.586–2.550	0.591
PCNA expression	2.806	1.740–4.527	<0.001	1.400	0.776–2.526	0.264

**Table 5:** GR serum level in colon adenocarcinoma patients.

	GR Serum Level in Colon Adenocarcinoma Patients								
	N	%	M	Me	Min	Max	Q1	Q3	SD
x ≤ Q1	16	25.00	6.83	4.82	1.98	19.03	2.78	11.00	5.52
Q1 < x < Q3	32	50.00	48.73	51.49	19.78	67.85	34.84	58.33	14.70
x ≥ Q3	16	25.00	85.85	86.75	76.41	98.16	78.94	90.57	7.95

## Data Availability

Data are contained within the article.

## References

[1] Lewandowska A., Rudzki G., Lewandowski T., Stryjkowska-Góra A., Rudzki S. (2022). Risk Factors for the Diagnosis of Colorectal Cancer. Cancer Control.

[2] Siegel R.L., Wagle N.S., Cercek A., Smith R.A., Jemal A. (2023). Colorectal cancer statistics, 2023. CA Cancer J. Clin..

[3] Stoffel E.M., Murphy C.C. (2020). Epidemiology and Mechanisms of the Increasing Incidence of Colon and Rectal Cancers in Young Adults. Gastroenterology.

[4] Healy M.A., Thirumurthi S., You Y.N. (2019). Screening high-risk populations for colon and rectal cancers. J. Surg. Oncol..

[5] Pino M.S., Chung D.C. (2010). The chromosomal instability pathway in colon cancer. Gastroenterology.

[6] Limoli C.L., Giedzinski E. (2003). Induction of chromosomal instability by chronic oxidative stress. Neoplasia.

[7] Mena S., Ortega A., Estrela J.M. (2009). Oxidative stress in environmental-induced carcinogenesis. Mutat. Res..

[8] Poprac P., Jomova K., Simunkova M., Kollar V., Rhodes C.J., Valko M. (2017). Targeting Free Radicals in Oxidative Stress-Related Human Diseases. Trends Pharmacol. Sci..

[9] Arnér E.S., Holmgren A. (2000). Physiological functions of thioredoxin and thioredoxin reductase. Eur. J. Biochem..

[10] Couto N., Wood J., Barber J. (2016). The role of glutathione reductase and related enzymes on cellular redox homoeostasis network. Free Radic. Biol. Med..

[11] Lu S.C. (2013). Glutathione synthesis. Biophys. Acta.

[12] Evans P., Halliwell B. (1999). Free radicals and hearing. Cause, consequence, and criteria. Ann. N. Y. Acad. Sci..

[13] Rogers L.K., Tamura T., Rogers B.J., Welty S.E., Hansen T.N., Smith C.V. (2004). Analyses of glutathione reductase hypomorphic mice indicate a genetic knockout. Toxicol. Sci..

[14] Kamerbeek N.M., van Zwieten R., de Boer M., Morren G., Vuil H., Bannink N., Lincke C., Dolman K.M., Becker K., Schirmer R.H. (2007). Molecular basis of glutathione reductase deficiency in human blood cells. Blood.

[15] Acevedo-León D., Monzó-Beltrán L., Gómez-Abril S.Á., Estañ-Capell N., Camarasa-Lillo N., Pérez-Ebri M.L., Escandón-Álvarez J., Alonso-Iglesias E., Santaolaria-Ayora M.L., Carbonell-Moncho A. (2021). The Effectiveness of Glutathione Redox Status as a Possible Tumor Marker in Colorectal Cancer. Int. J. Mol. Sci..

[16] Moghadamyeghaneh Z., Hanna M.H., Carmichael J.C., Mills S.D., Pigazzi A., Stamos M.J. (2015). Preoperative leukocytosis in colorectal cancer patients. J. Am. Coll. Surg..

[17] Zeng J., Tang Z.H., Liu S., Guo S.S. (2017). Clinicopathological significance of overexpression of interleukin-6 in colorectal cancer. World J. Gastroenterol..

[18] Jagust P., Alcalá S., Sainz Jr B., Heeschen C., Sancho P. (2020). Glutathione metabolism is essential for self-renewal and chemoresistance of pancreatic cancer stem cells. World J. Stem Cells.

[19] Abdel Hadi N., Reyes-Castellanos G., Carrier A. (2021). Targeting Redox Metabolism in Pancreatic Cancer. Int. J. Mol. Sci..

[20] Nishizawa S., Araki H., Ishikawa Y., Kitazawa S., Hata A., Soga T., Hara T. (2018). Low tumor glutathione level as a sensitivity marker for glutamate-cysteine ligase inhibitors. Oncol. Lett..

[21] Kim A.D., Zhang R., Han X., Kang K.A., Piao M.J., Maeng Y.H., Chang W.Y., Hyun J.W. (2015). Involvement of glutathione and glutathione metabolizing enzymes in human colorectal cancer cell lines and tissues. Mol. Med. Rep..

[22] Maffei F., Angeloni C., Malaguti M., Moraga J.M., Pasqui F., Poli C., Colecchia A., Festi D., Hrelia P., Hrelia S. (2011). Plasma antioxidant enzymes and clastogenic factors as possible biomarkers of colorectal cancer risk. Mutat. Res..

[23] Wu R., Feng J., Yang Y., Dai C., Lu A., Li J., Liao Y., Xiang M., Huang Q., Wang D. (2017). Significance of Serum Total Oxidant/Antioxidant Status in Patients with Colorectal Cancer. PLoS ONE..

[24] Cecerska-Heryć E., Surowska O., Heryć R., Serwin N., Napiontek-Balińska S., Dołęgowska B. (2021). Are antioxidant enzymes essential markers in the diagnosis and monitoring of cancer patients—A review. Clin. Biochem..

[25] Gopčević K.R., Rovčanin B.R., Tatić S.B., Krivokapić Z.V., Gajić M.M., Dragutinović V.V. (2013). Activity of superoxide dismutase, catalase, glutathione peroxidase, and glutathione reductase in different stages of colorectal carcinoma. Dig. Dis. Sci..

[26] Skrzydlewska E., Sulkowski S., Koda M., Zalewski B., Kanczuga-Koda L., Sulkowska M. (2005). Lipid peroxidation and antioxidant status in colorectal cancer. World J. Gastroenterol..

[27] Skrzydlewska E., Stankiewicz A., Sulkowska M., Sulkowski S., Kasacka I. (2001). Antioxidant status and lipid peroxidation in colorectal cancer. J. Toxicol. Environ. Health A.

[28] Zińczuk J., Maciejczyk M., Zaręba K., Romaniuk W., Markowski A., Kędra B., Zalewska A., Pryczynicz A., Matowicka-Karna J., Guzińska-Ustymowicz K. (2019). Antioxidant Barrier, Redox Status, and Oxidative Damage to Biomolecules in Patients with Colorectal Cancer. Can Malondialdehyde and Catalase Be Markers of Colorectal Cancer Advancement?. Biomolecules.

[29] Gaya-Bover A., Hernández-López R., Alorda-Clara M., Ibarra de la Rosa J.M., Falcó E., Fernández T., Company M.M., Torrens-Mas M., Roca P., Oliver J. (2020). Antioxidant enzymes change in different non-metastatic stages in tumoral and peritumoral tissues of colorectal cancer. Int. J. Biochem. Cell Biol..

[30] Strzelczyk J.K., Wielkoszyński T., Krakowczyk Ł., Adamek B., Zalewska-Ziob M., Gawron K., Kasperczyk J., Wiczkowski A. (2012). The activity of antioxidant enzymes in colorectal adenocarcinoma and corresponding normal mucosa. Acta Biochim. Pol..

[31] Brzozowa-Zasada M., Piecuch A., Michalski M., Matysiak N., Kucharzewski M., Łos M.J. (2023). The Clinical Application of Immunohistochemical Expression of Notch4 Protein in Patients with Colon Adenocarcinoma. Int. J. Mol. Sci..

[32] Brzozowa-Zasada M., Ianaro A., Piecuch A., Michalski M., Matysiak N., Stęplewska K. (2023). Immunohistochemical Expression of Glutathione Peroxidase-2 (Gpx-2) and Its Clinical Relevance in Colon Adenocarcinoma Patients. Int. J. Mol. Sci..

[33] Frithiof H., Welinder C., Larsson A.M., Rydén L., Aaltonen K. (2015). A novel method for downstream characterization of breast cancer circulating tumor cells following CellSearch isolation. J. Transl. Med..

[34] Assi M. (2017). The differential role of reactive oxygen species in early and late stages of cancer. Am. J. Physiol. Regul. Integr. Comp. Physiol..

[35] Jones D.P. (2008). Radical-free biology of oxidative stress. Am. J. Physiol.-Cell Physiol..

[36] Federico A., Morgillo F., Tuccillo C., Ciardiello F., Loguercio C. (2007). Chronic inflammation and oxidative stress in human carcinogenesis. Int. J. Cancer.

[37] Perse M. (2013). Oxidative stress in the pathogenesis of colorectal cancer: Cause or consequence?. Biomed. Res. Int..

[38] Aboushousha R., van der Velden J., Hamilton N., Peng Z., MacPherson M., Erickson C., White S., Wouters E.F.M., Reynaert N.L., Seward D.J. (2023). Glutaredoxin attenuates glutathione levels via deglutathionylation of Otub1 and subsequent destabilization of system xC. Sci Adv..

[39] Kennedy L., Sandhu J.K., Harper M.E., Cuperlovic-Culf M. (2020). Role of Glutathione in Cancer: From Mechanisms to Therapies. Biomolecules.

[40] Zhao Y., Seefeldt T., Chen W., Carlson L., Stoebner A., Hanson S., Foll R., Matthees D.P., Palakurthi S., Guan X. (2009). Increase in thiol oxidative stress via glutathione reductase inhibition as a novel approach to enhance cancer sensitivity to X-ray irradiation. Free Radic. Biol. Med..

[41] Townsend D.M., Tew K.D., Tapiero H. (2003). The importance of glutathione in human disease. Biomed. Pharmacother..

[42] Wu G., Fang Y.Z., Yang S., Lupton J.R., Turner N.D. (2004). Glutathione metabolism and its implications for health. J. Nutr..

[43] de Souza L.F., Schmitz A.E., da Silva L.C.S., de Oliveira K.A., Nedel C.B., Tasca C.I., de Bem A.F., Farina M., Dafre A.L. (2017). Inhibition of reductase systems by 2-AAPA modulates peroxiredoxin oxidation and mitochondrial function in A172 glioblastoma cells. Toxicol. In Vitro.

[44] Li X., Jiang Z., Feng J., Zhang X., Wu J., Chen W. (2017). 2-Acetylamino-3-[4-(2-acetylamino-2-carboxyethylsulfanylcarbonylamino) phenyl carbamoylsulfanyl] propionic acid, a glutathione reductase inhibitor, induces G2/M cell cycle arrest through generation of thiol oxidative stress in human esophageal cancer cells. Oncotarget.

[45] Li X., Wu J., Zhang X., Chen W. (2018). Glutathione reductase-mediated thiol oxidative stress suppresses metastasis of murine melanoma cells. Free Radic. Biol. Med..

[46] Piecuch A., Kurek J., Kucharzewski M., Wyrobiec G., Jasiński D., Brzozowa-Zasada M. (2020). Catalase immunoexpression in colorectal lesions. Prz. Gastroenterol..

[47] Piecuch A., Brzozowa-Zasada M., Dziewit B., Segiet O., Kurek J., Kowalczyk-Ziomek G., Wojnicz R., Helewski K. (2016). Immunohistochemical assessment of mitochondrial superoxide dismutase (MnSOD) in colorectal premalignant and malignant lesions. Prz. Gastroenterol..

[48] Nozoe T., Honda M., Inutsuka S., Yasuda M., Korenaga D. (2003). Significance of immunohistochemical expression of manganese superoxide dismutase as a marker of malignant potential in colorectal carcinoma. Oncol. Rep..

[49] Brzozowa-Zasada M., Piecuch A., Bajdak-Rusinek K., Janelt K., Michalski M., Klymenko O., Matysiak N. (2023). Immunohistochemical Expression of Glutathione Peroxidase 1 (Gpx-1) as an Independent Prognostic Factor in Colon Adenocarcinoma Patients. Pharmaceuticals.

[50] Robbins D., Zhao Y. (2014). Manganese superoxide dismutase in cancer prevention. Antioxid Redox Signal..

[51] Chandel N.S. (2010). Mitochondrial regulation of oxygen sensing. Adv. Exp. Med. Biol..

[52] Preci D.P., Almeida A., Weiler A.L., Mukai Franciosi M.L., Cardoso A.M. (2021). Oxidative damage and antioxidants in cervical cancer. Int. J. Gynecol. Cancer.

[53] Prat A., Karginova O., Parker J.S., Fan C., He X., Bixby L., Harrell J.C., Roman E., Adamo B., Troester M. (2013). Characterization of cell lines derived from breast cancers and normal mammary tissues for the study of the intrinsic molecular subtypes. Breast Cancer Res. Treat..

[54] Ertel A., Verghese A., Byers S.W., Ochs M., Tozeren A. (2006). Pathway-specific differences between tumor cell lines and normal and tumor tissue cells. Mol. Cancer..

[55] Gostimskaya I., Grant C.M. (2016). Yeast mitochondrial glutathione is an essential antioxidant with mitochondrial thioredoxin providing a back-up system. Free Radic. Biol. Med..

[56] Go Y.M., Jones D.P. (2010). Redox control systems in the nucleus: Mechanisms and functions. Antioxid. Redox Signal..

[57] Longley D.B., Harkin D.P., Johnston P.G. (2003). 5-fluorouracil: Mechanisms of action and clinical strategies. Nat. Rev. Cancer.

[58] Santiago-Arteche R., Muñiz P., Cavia-Saiz M., Garcia-Giron C., García-Gonzalez M., Llorente-Ayala B., Corral M.J. (2012). Cancer chemotherapy reduces plasma total polyphenols and total antioxidants capacity in colorectal cancer patients. Mol. Biol. Rep..

[59] Simone C.B., Simone N.L., Simone V., Simone C.B. (2007). Antioxidants and other nutrients do not interfere with chemotherapy or radiation therapy and can increase kill and increase survival, part 1. Altern. Ther. Health Med..

[60] Jiang H., Zuo J., Li B., Chen R., Luo K., Xiang X., Lu S., Huang C., Liu L., Tang J. (2023). Drug-induced oxidative stress in cancer treatments: Angel or devil?. Redox Biol..

[61] Xiong Y., Xiao C., Li Z., Yang X. (2021). Engineering nanomedicine for glutathione depletion-augmented cancer therapy. Chem. Soc. Rev..

[62] Tormos C., Javier Chaves F., Garcia M.J., Garrido F., Jover R., O’Connor J.E., Iradi A., Oltra A., Oliva M.R., Sáez G.T. (2004). Role of glutathione in the induction of apoptosis and c-fos and c-jun mRNAs by oxidative stress in tumor cells. Cancer Lett..

[63] Chiang F.F., Huang S.C., Yu P.T., Chao T.H., Huang Y.C. (2023). Oxidative Stress Induced by Chemotherapy: Evaluation of Glutathione and Its Related Antioxidant Enzyme Dynamics in Patients with Colorectal Cancer. Nutrients.

[64] Gao P., Zhang H., Dinavahi R., Li F., Xiang Y., Raman V., Bhujwalla Z.M., Felsher D.W., Cheng L., Pevsner J. (2007). HIF-dependent antitumorigenic effect of antioxidants in vivo. Cancer Cell.

[65] Piskounova E., Agathocleous M., Murphy M.M., Hu Z., Huddlestun S.E., Zhao Z., Leitch A.M., Johnson T.M., DeBerardinis R.J., Morrison S.J. (2015). Oxidative stress inhibits distant metastasis by human melanoma cells. Nature.

[66] Glasauer A., Chandel N.S. (2014). Targeting antioxidants for cancer therapy. Biochem. Pharmacol..

